# Neuroprotective and Neuroregenerative Effects of Nimodipine in a Model System of Neuronal Differentiation and Neurite Outgrowth

**DOI:** 10.3390/molecules20011003

**Published:** 2015-01-09

**Authors:** Kaya Bork, Franziska Wurm, Hannes Haller, Christian Strauss, Christian Scheller, Vinayaga S. Gnanapragassam, Rüdiger Horstkorte

**Affiliations:** 1 Institute for Physiological Chemistry, Martin-Luther University, Hollystr. 1, Halle (Saale) D-06114, Germany; E-Mails: franziska.wurm@googlemail.com (F.W.); hannes.haller85@gmail.com (H.H.); vinayaga.gnanapragassam@medizin.uni-halle.de (V.S.G.); ruediger.horstkorte@medizin.uni-halle.de (R.H.); 2 Department of Neurosurgery, Martin-Luther-University Halle-Wittenberg, Ernst-Grube-Straße 40, Halle (Saale) D-06120, Germany; E-Mails: christian.strauss@medizin.uni-halle.de (C.St.); christian.scheller@medizin.uni-halle.de (C.Sc.)

**Keywords:** Ca^2+^ channels, neuroprotection, neurite outgrowth, nimodipine

## Abstract

Nimodipine is a Ca^2+^-channel antagonist mainly used for the management of aneurysmal subarachnoid hemorrhage (aSAH) to prevent cerebral vasospasms. However, it is not clear if the better outcome of nimodipine-treated patients is mainly due to vasodilatation or whether other cellular neuroprotective or neuregenerative effects of nimodipine are involved. We analysed PC12 cells after different stress stimuli with or without nimodipine pretreatment. Cytotoxicity of 200 mM EtOH and osmotic stress (450 mosmol/L) was significantly reduced with nimodipine pretreatment, while nimodipine has no influence on the hypoxia-induced cytotoxicity in PC12 cells. The presence of nimodipine also increased the NGF-induced neurite outgrowth in PC12 cells. However, nimodipine alone was not able to induce neurite outgrowth in PC12 cells. These results support the idea that nimodipine has general neuroprotective or neuregenerative effect beside its role in vasodilatation and is maybe useful also in other clinical applications beside aSAH.

## 1. Introduction

**Figure 1:**
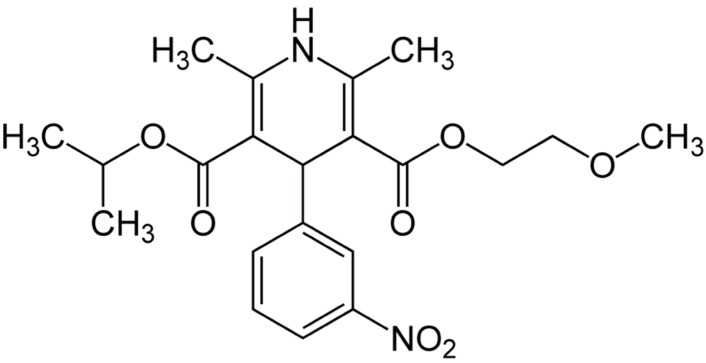
Chemical structure of nimodipine.

Nimodipine (Figure 1) is a 1,4-dihydropyridine l-type-Ca^2+^-channel antagonist. It is a more lipophilic analog of nifedipine and easily crosses the blood brain barrier and reaches high concentrations in cerebrospinal fluid (CSF) [1]. It has been shown that nimodipine dilates cerebral vessels and improves cerebral blood flow at doses that do not affect the dilatation of peripheral blood vessels and the systemic arterial pressure [2]. Therefore nimodipine is mainly used for the management of aneurysmal subarachnoid hemorrhage (aSAH) and has a proven effect in reducing poor outcome following aSAH [3]. However, nimodipine has been evaluated in a variety of other applications. During ageing, the control of the intracellular calcium concentration is impaired and this has been associated with age-related neurodegenerative conditions [4,5]. This led to clinical trials where dementia patients were treated with nimodipine. They showed some benefit for patients with Alzheimer's disease and cerebrovascular disease [6]. Nimodipine also has beneficial effects in the regeneration process after skull base, laryngeal and maxillofacial surgery in several animal experiments [7,8,9] and clinical series [10,11,12]. However, it is not clear if the better outcome of nimodipine treated patients is mainly due to vasodilatation or if other neuroprotective or neuregenerative effects of nimodipine play a role [2].

In recent years the neuroprotective potential of nimodipine for different noxious agents, which are associated with neurotoxicity has been analyzed. In a rat model of chronic alcoholic encelphalopathy nimodipine showed protective effects against alcohol-induced cerebrovascular damage [13]. Methylmercury (MeHg) is a neurotoxicant and exposure results primarily in sensory and motor deficits [14]. Dietary nimodipine delayed or prevented MeHg-induced behavioral toxicity in mice [15].

In the present study we analyzed the neuroprotective or neuroregenerative potential of nimodipine in the rat adrenal pheochromocytoma cell line PC12. This cell line is commonly used as a model system for neuronal differentiation, neurite outgrowth and neurotoxicological studies [16,17].

## 2. Results and Discussion

### 2.1. Results

We analyzed the neuroprotective or neurogenerative potential of nimodipine in PC12 cells. PC12 cells have been used for many years as a model neuronal-like cell-line [16]) and commonly been used to model neuronal stress. The neuroprotective effect of nimodipine was investigated under different stress conditions. We induced alcohol, osmotic and hypoxic stress in PC12 cells. Under all stress conditions 5 × 10^5^ cells were cultured in the absence or presence of 20 µM nimodipine 48 h prior and during the stress experiment. Pretreatment with nimodipine was included because positive effects were described in some clinical trials [11]. The stress-induced cytotoxicity was measured by lactate dehydrogenase (LDH) release to the medium as described in the methods.

**Figure 2:**
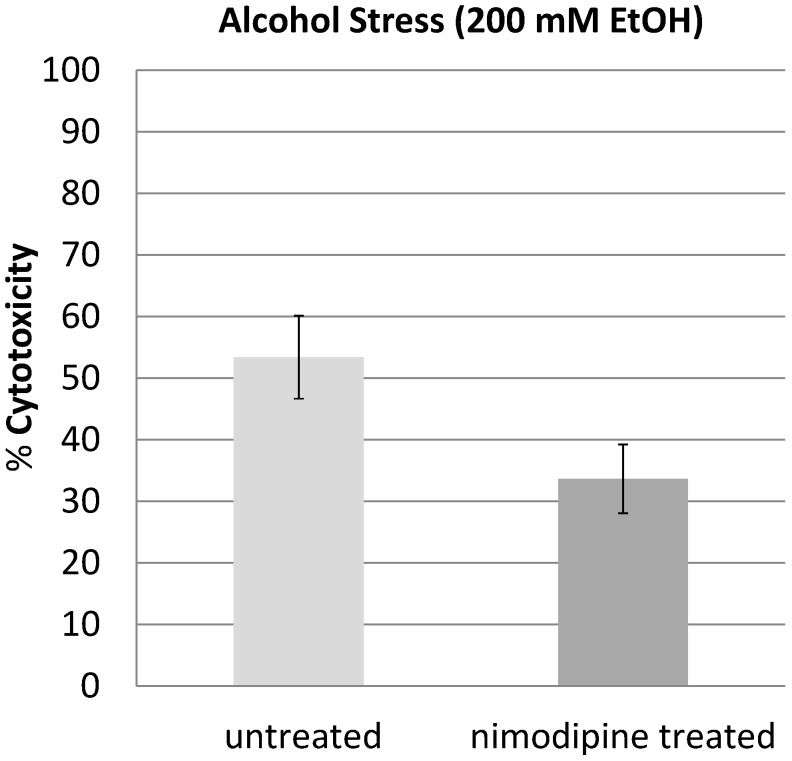
PC12 cells were treated with 200 mM EtOH and 20 μM nimodipine or DMSO and incubated for 48 h. The EtOH induced cytotoxicity of 53.4% in the untreated PC12 was reduced to 33.7% cytotoxicity in nimodipine-treated cultures.

To investigate ethanol-induced cytotoxicity, cells were treated with 200 mM ethanol for 48 h. The result was a cytotoxicity of 53.4% compared with 33.7% cytotoxicity in nimodipine-treated cultures. This was a significantly reduced ethanol-induced cytotoxicity by 39.1% (*p <* 0.05) (Figure 2).

**Figure 3:**
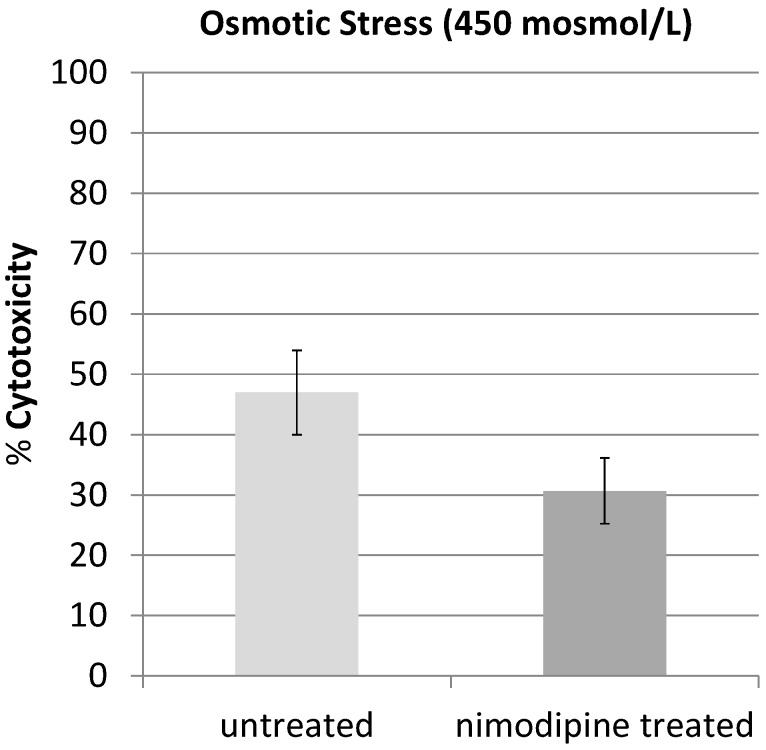
PC12 cells were treated with additional NaCl (450 mosmol/L) and 20 µM nimodipine or DMSO and incubated for 48 h. The osmotic stress induced cytotoxicity of 47% in the untreated PC12 was reduced to 30.7% cytotoxicity in nimodipine-treated cultures.

In a next set of experiments, we induced osmotic stress. Therefore, cells were treated with additional NaCl to increase the osmotic concentration to 450 mosmol/L for 48 h. In comparison to untreated cells that had 47% cytotoxicity, nimodipine treated cells had significantly (*p <* 0.05) reduced 30.7% cytotoxicity (Figure 3). The treatment with nimodopine reduced the osmotic stress cytotoxicity by 34.7%.

**Figure 4:**
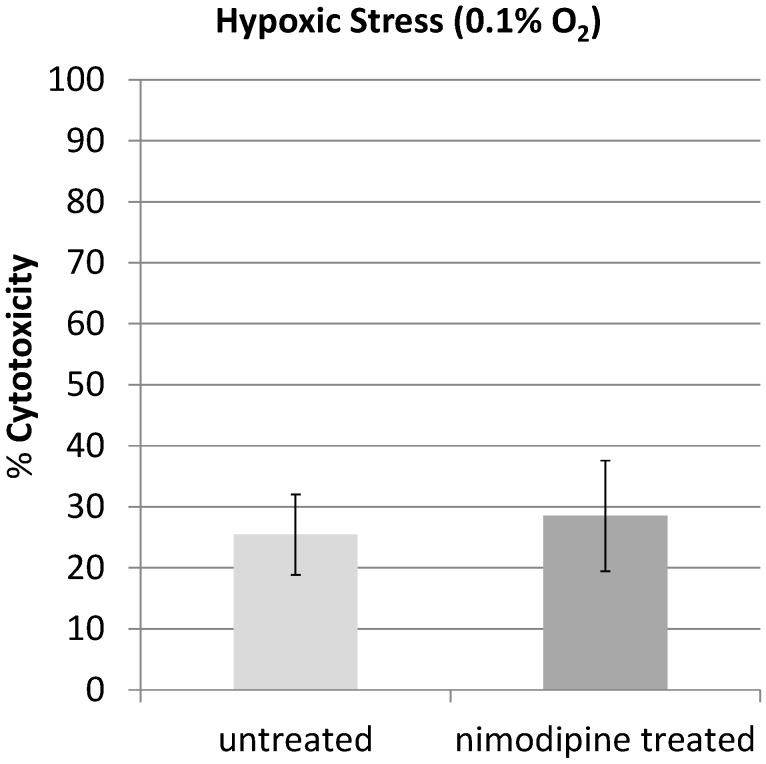
PC12 cells were cultivated under 0.1% O_2_ for 3 h to induce hypoxic stress. No significant difference in the cytotoxicity between nimodipine-treated (25.4%) and untreated (28.5%) PC12 cells could be measured.

We then cultivated PC12 cells under 0.1% O_2_ for 3 h to induce hypoxic stress. Under these conditions, no significant difference in the LDH-release between nimodipine-treated and untreated PC12 cells could be measured (Figure 4). Also longer incubation periods to up to 8 h did not show any effects of nimodipine on hypoxia-induced cytotoxicity (data not shown).

**Figure 5:**
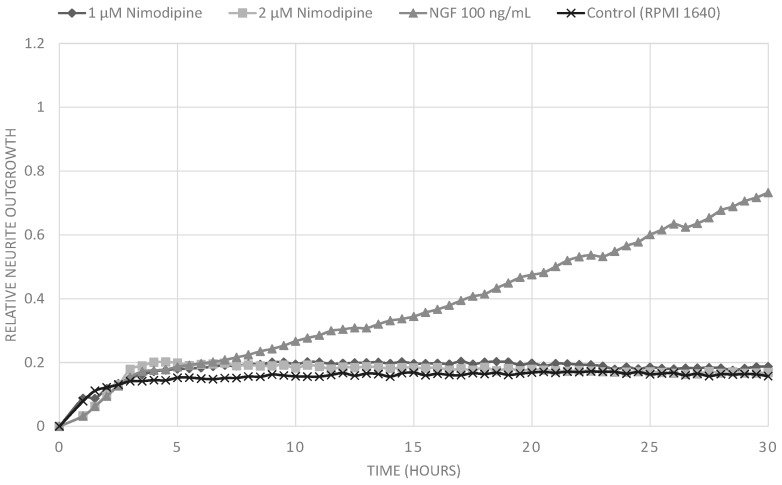
Measurement of realtive neurite outgrowth of PC12 cells using the xCELLigence real-time cell analyzer. Cells were treated with with 100 ng/mL NGF, 1 µM or 2 µM nimodipine. Untreated cells or cells treated with 1 µM or 2 µM nimodipine showed no neurite outgrowth, while treatment with 100 ng/mL NGF led to neurite outgrowth.

**Figure 6:**
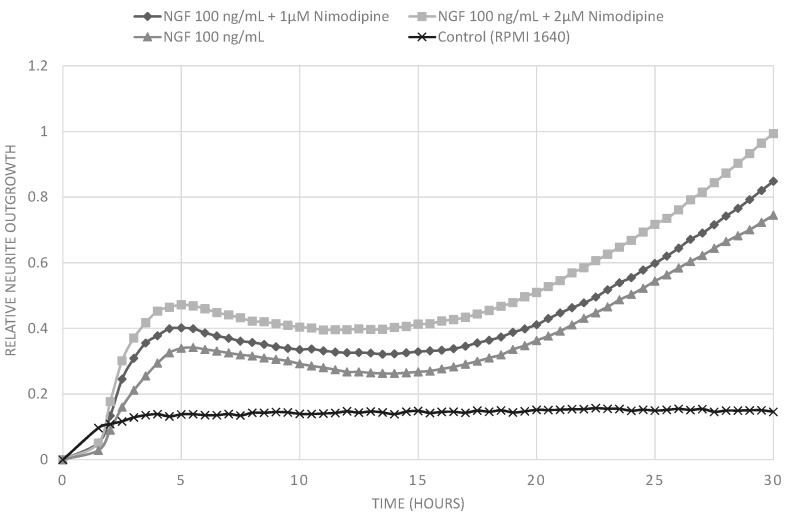
Measurement of realtive neurite outgrowth of PC12 cells using the xCELLigence real-time cell analyzer. Cells were treated with 100 ng/mL NGF alone or in combination with 1 µM or 2 µM nimodipine. Additionally treatment with 1 µM or 2 µM nimodipine led to dose dependent increase in of NGF induced neurite outgrowth.

Treatment of PC12 cells with nerve growth factor (NGF) leads to neurite outgrowth and neuronal differentiation. We analyzed the effect of nimodipine on the NGF-induced neuronal differentiation of P12 cells to evaluate its neuroregenerative potential. We established a rapid measurement for neurite outgrowth in a real-time cell analyzer (xCELLLigence). The xCELLigence system quantifies cell growth and morphology in real time in cell culture. Untreated cells or cells cultured only in the presence of 1 µM or 2 µM nimodipine did not extend neurites at all. Culturing PC12 cells in the presence of 100 ng/mL NGF resulted in substantial neurite outgrowth (Figure 5). However, NGF-induced neurite outgrowth was increased by additional treatment with 1 µM or 2 µM nimodipine in a concentration-dependent manner (Figure 6). In this experiments lower concentrations of nimodipine were applied because clinical trials with dementia patients used lower dosis of nimodipine over a long periode, while studys focusing on neuroprotection using higher dosis of nimodipine over a short period [18].

**Figure 7:**
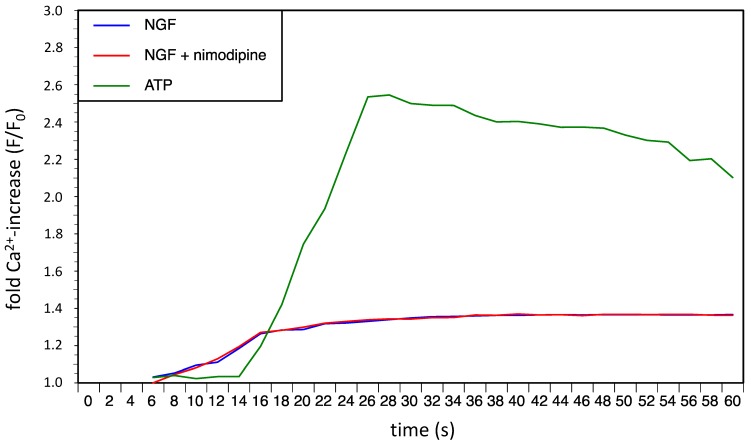
Fold Ca^2+^-increase (F/F_0_) in PC12 cells after stimulus with NGF (100 ng/mL) alone or NGF (100 ng/mL) in combination with nimodipine (2 µM). ATP (150 µM) was used as a positive control for intracellular calcium mobilization.

Nimodipine is a Ca^2+^-channel antagonist that could influence the NGF induced calcium response in PC12 cells. Therefore we analyzed the NGF induced initial intracellular calcium mobilization with and without 2 µM nimodipine by fluorescent probes (FluoForte). The typical rapid but low NGF induced initial intracellular calcium mobilization showed no difference in presence of 2 µM nimodipine (Figure 7).

We also analyzed the influence of 1 µM and 2 µM nimodipine on the proliferation of PC12 cells using a standard bromodeoxyuridine (BrdU) based assay. Neither nimodipine alone nor nimodipine in combination with 100 ng/mL NGF (compared with 100 ng/mL NGF alone) showed any significant effects on the proliferation of PC12 cells (data not shown).

### 2.2. Discussion

The 1,4-dihydropyridine l-type-Ca^2+^-channel antagonist nimodipine is mainly used for the management of aneurysmal subarachnoid hemorrhage (aSAH). However, in recent years the neuroprotective effect of nimodipine has been evaluated in different other settings like treatment of dementia or to improve the regeneration process after skull base, laryngeal and maxillofacial surgery. If the better outcome of nimodipine treated aSAH-patients is due to reduced vasospasm and improvement of cerebral blood flow by nimodipine induced vasodilation or if other neuroprotective effects of nimodipine play a role is still discussed [19]. Overload of intracellular Ca^2+^ by massive glutamate release during cerebral ischemia is known to contribute to neuronal death and in general activation of l-type Ca^2+^ channel represents a critical step towards neurodegeneration. In the present study, we showed that pretreatment with nimodipine reduces alcohol (200 mM EtOH) and osmotic stress (450 mosmol/L) induced cytotoxicity in PC12-cells significantly. In our experiments pretreatment with nimodipine showed no effect on hypoxic stress induced by cultivating PC12 cells under 0.1% O_2_ for 3 h or longer. Lecht *et al.* described that nimodipine treatment reduces cytotoxicity in PC12-cells induced by oxygen-glucose deprivation (OGD) [20]. However, in the OGD experiments the PC12-cells were cultivated only under milder conditions (1% O_2_) and the protective effect of nimodipine was limited to the first 5 h of OGD treatment. This indicates that nimodipine is most effective at milder stress conditions. It should be considered in future clinical trials that the neuroprotective effect of nimpodipine could depend on the degree of the neurological damage. We showed here that the nimodipine enhances the NGF induced neurite outgrowth in PC12 cells. Neurite outgrowth depends on optimal levels of intracellular Ca^2+^. A change in the NGF induced initial intracellular calcium mobilization in the presence of nimodipine could not be observed in our experiments. However, waves of calcium currents located in the growth cones are vital for the regulation of NGF induced neurite outgrowth in PC12 cells [21] and changing l-type Ca^2+^ channel distribution was observed in this process [22]. Variances in this growth cone located calcium currents by nimodipine could lead to the observed changes in neurite outgrowth. This goes along with the observation that combinations of NGF and nimodipine have positive effects in rat sciatic nerve injury regeneration [23] and that nimodipine can accelerate the axonal regeneration following peripheral nerve injury in rats [8].

## 3. Experimental Section

### 3.1. Cell Line

PC12 cells were obtained from the Leibniz Institute DSMZ-German Collection of Microorganisms and Cell Cultures GmbH (Braunschweig, Germany).

### 3.2. Cell Culture

PC12 cells were cultured in Falcon plastic flasks in RPMI-1640 supplemented with 10% horse serum (Seromed, Wien, Austria) under a humidified atmosphere with 20% O_2_ and 5% CO_2_ at 37 °C.

### 3.3. Stress Assays

For assays, 5 × 10^5^ cells were seed on Collagen IV coated plates (10 cm) and allowed to attach for 24 h. Afterwards cells were pretreated with 20 μM nimodipine or DMSO for 48 h.

Alcohol stress: Medium was changed and cells were treated with 200 mM EtOH and 20 µM nimodipine or DMSO and incubated for 48 h.

Osmotic stress: Medium was changed and cells were treated with additional NaCl to increase the osmotic concentration from 290 to 450 mosmol/L and 20 µM nimodipine or DMSO and incubated for 48 h.

Hypoxia: Cells were cultivated under 0.1% O_2_ for 3–8 h using an Invivo_2_ 400 hypoxia workstation (Ruskinn, Sanford, ME, USA) attached to a Ruskinn gas mixer Q.

### 3.4. LDH-Assay

As a general measure of cell death, LDH activity was measured in samples of culture medium collected after stress-assays according to the method of Wroblewski and LaDue [24]. In brief, samples of media (40 µL) were added to 80 µL reaction buffer (2.3 µmol of sodium pyruvate and 111 µg NADH added in 0.1 M KPO_4_ buffer (pH 7.5 at 25 °C)). The absorbance of the reaction mixture at 340 nm, an index of NADH concentration, was recorded at 2 s intervals using a GeneSys 10 Bio spectrophotometer from Thermo Scientific (Schwerte, Germany). LDH concentration was calculated from the slope of the absorbance curve, fit by linear regression to the linear (initial) portion of the curve. Accuracy of the assay was verified by periodic checks of a standard LDH enzyme solution (Enzyme Control 2-E, Sigma, St. Louis, MO, USA). To calculate the cytotoxicity 5 × 10^5^ PC12 cells were lysed by adding 1% Triton X-100 to the medium and the LDH concentration was measured and set to 100% cytotoxicity.

### 3.5. Neurite Outgrowth

PC12 cells were routinely seeded on E-plates (Roche, Penzberg, Germany) at a density of 100 cells/mm^2^. Cells were cultured for up to 48 h in the absence or presence of 100 ng NGF, 1 µM or 2 µM nimodipine or both. Neurite outgrowth of the cultures was continuously analyzed by the xCELLigence system (Roche) via electrical impedance changes on the gold coated E-plates induced by cell shape variances. We have previously shown that the xCELLigence system suitable for quantification of neurite outgrowth in PC12 by comparing it to a conventional method [25].

### 3.6. Calcium-Assay

For the intracellular calcium-assays we used the FluoFort calcium assay kit from Enzo Life Science (Farmingdale, NY, USA). The assay was performed according to the manual. In brief: PC12 cells were resuspended FluoFort dye-loading solution (Hanks Balanced Salt Solution, dye efflux inhibitor, FluoFort dye). Cells were plated using 2.5 × 105 cells per 96-well plates well at a plating volume of 100 µL per well and incubated for 45 min at 37 °C. NGF (100 ng/mL) and nimodipine (2 µM) was added directly prior measurement. Positive control was induced by adding 150 µM ATP and DMSO was added to the baseline control. The fluorescence values were recorded with a time resolution of 3 s using a Fluoroskan Ascent from Labsystems (Helsinki, Finnland) (Ex = 485 nm/Em = 530 nm). Recording was started 5 s after adding the stimulus reagents.

### 3.7. Cell Proliferation Assay

We used the Cell Proliferation Assay from Calbiochem (San Diego, CA, USA) according to the manual. In brief: 2 × 10^4^ PC12 cells per well were seed into a 96 well culture dish and BrdU was added. Cells were cultivated for 12 or 24 h in the presence of 100 ng NGF, 1 µM or 2 µM Nimodipine or both. Afterwards cells were fixed and incubated with an Anti-BrdU-Antibody. Cells were stained using a secondary HRP coupled antibody and tetra-methylbenzidine. Staining was quantified using a Multiskan EX (Thermo Fisher) at 450 nm.

### 3.8. Statistical Analysis

Data from the stress assay were calculated from at least 10 independent experiments. Statistical analysis was carried out using the unpaired *t*-test.

## 4. Conclusions

In summary, our data indicate that nimodipine has a neuroprotective capability concerning some types of stressors and enhance the NGF induced neurite outgrowth in PC12 cells. This supports the idea that nimodipine is a candidate to be used as a therapeutic for neurodegenerative diseases and as preventive medication in neurosurgery. However, the detailed mode of action of nimodipine as a neuroprotective and neuroregeneration promoting substance remain unknown and need to be addressed in further studies to foster its application and therapy options.

## Supplementary Materials

Supplementary materials can be accessed at: http://www.mdpi.com/1420-3049/20/01/1003/s1.
