# Supplementary material for: Neuroprotective and Neuroregenerative Effects of Nimodipine in a Model System of Neuronal Differentiation and Neurite Outgrowth
Source: Molecules. 2015 Jan 9;20(1):1003–13. doi: 10.3390/molecules20011003 (PMC6272420; doi:10.3390/molecules20011003)
Supplement: Supplementary file 1 [file molecules-20-01003-s001.pdf]

Supplementary Materials

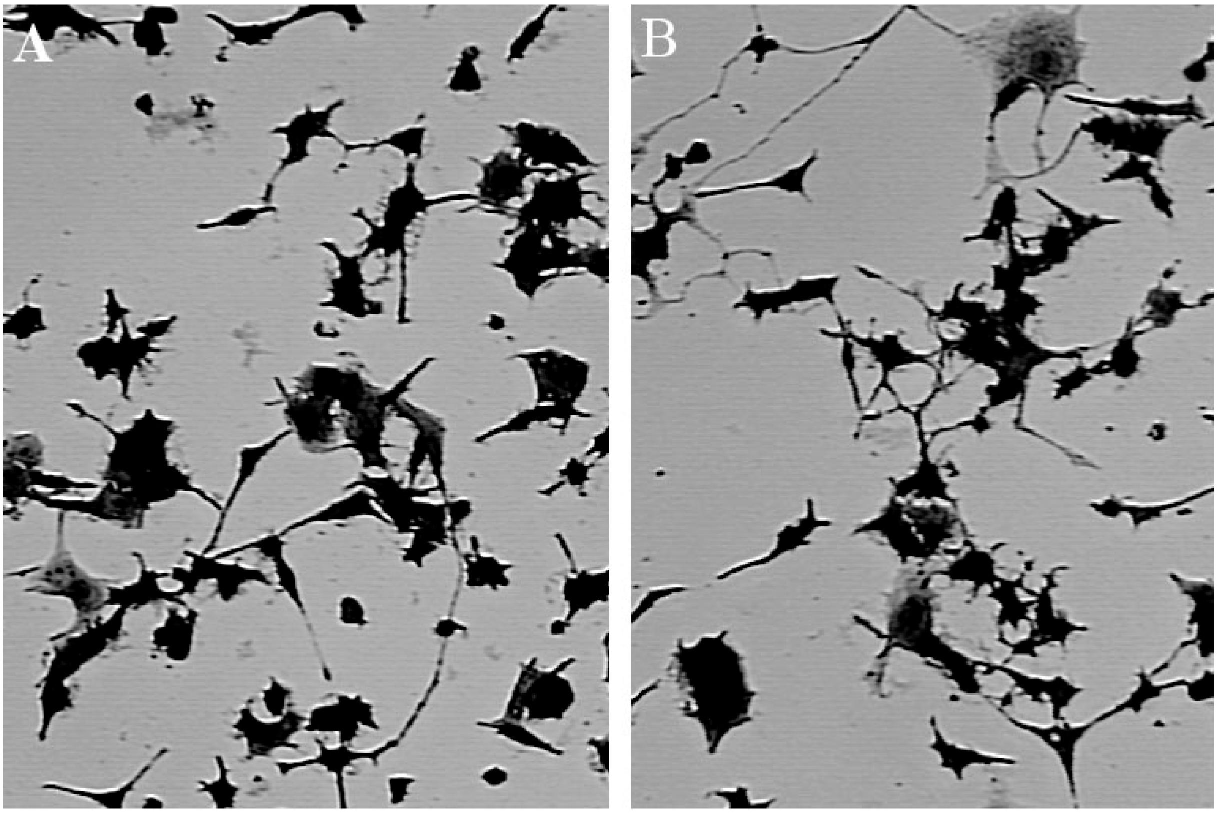

Figure S1. Representative images for neurite outgrowth of PC12 cells. PC12 cells stained with crystal violet after treatment with 100 ng/mL NGF (**A**) or 100 ng/mL NGF and 2 μM nimodipine (**B**) for 20 h.
